# Preventing Oxidative Stress in the Liver: An Opportunity for GLP-1 and/or PASK

**DOI:** 10.3390/antiox10122028

**Published:** 2021-12-20

**Authors:** Verónica Hurtado-Carneiro, Pilar Dongil, Ana Pérez-García, Elvira Álvarez, Carmen Sanz

**Affiliations:** 1 Department of Physiology, Faculty of Medicine, Institute of Medical Research at the San Carlos Clinic Hospital (IdISSC), Complutense University of Madrid, Ciudad Universitaria, 28040 Madrid, Spain; 2 Department of Biochemistry and Molecular Biology, Faculty of Medicine, Institute of Medical Research at the San Carlos Clinic Hospital (IdISSC), Complutense University of Madrid, Ciudad Universitaria, 28040 Madrid, Spain; pilar.dongil@uam.es (P.D.); anpere07@ucm.es (A.P.-G.); eao513@ucm.es (E.Á.); 3 Department of Cell Biology, Faculty of Medicine, Institute of Medical Research at the San Carlos Clinic Hospital (IdISSC), Complutense University of Madrid, Ciudad Universitaria, 28040 Madrid, Spain; mcsanz@med.ucm.es

**Keywords:** exendin-4, metabolic sensors, antioxidants

## Abstract

The liver’s high metabolic activity and detoxification functions generate reactive oxygen species, mainly through oxidative phosphorylation in the mitochondria of hepatocytes. In contrast, it also has a potent antioxidant mechanism for counterbalancing the oxidant’s effect and relieving oxidative stress. PAS kinase (PASK) is a serine/threonine kinase containing an N-terminal Per-Arnt-Sim (PAS) domain, able to detect redox state. During fasting/feeding changes, PASK regulates the expression and activation of critical liver proteins involved in carbohydrate and lipid metabolism and mitochondrial biogenesis. Interestingly, the functional inactivation of PASK prevents the development of a high-fat diet (HFD)-induced obesity and diabetes. In addition, PASK deficiency alters the activity of other nutrient sensors, such as the AMP-activated protein kinase (AMPK) and the mammalian target of rapamycin (mTOR). In addition to the expression and subcellular localization of nicotinamide-dependent histone deacetylases (SIRTs). This review focuses on the relationship between oxidative stress, PASK, and other nutrient sensors, updating the limited knowledge on the role of PASK in the antioxidant response. We also comment on glucagon-like peptide 1 (GLP-1) and its collaboration with PASK in preventing the damage associated with hepatic oxidative stress. The current knowledge would suggest that PASK inhibition and/or exendin-4 treatment, especially under fasting conditions, could ameliorate disorders associated with excess oxidative stress.

## 1. Introduction

The liver is a vital organ for adapting to nutritional changes (e.g., fasting/feeding states) by responding appropriately to achieve metabolic and energy homeostasis through its role in the storage and redistribution of carbohydrates, proteins, vitamins, and lipids.

## 2. Liver Metabolic Functions and Detoxification

After food intake, the liver stores glucose as glycogen, facilitating glycemic control [1]. Furthermore, the excess carbohydrate in carbohydrate-rich diets is converted into fatty acids via *de novo* lipogenesis [2,3].

By contrast, the liver produces glucose under fasting conditions, first by glycogenolysis and subsequently through hepatic gluconeogenesis, as the main fuel source for other tissues and contributing to whole-body energy homeostasis [3,4]. The liver’s high metabolic rate means it is also an important source of reactive oxygen species (ROS).

The liver is also the main organ involved in the detoxification of substances harmful to the body. Many drugs, various endogenous molecules, and xenobiotics are lipophilic molecules that need to be metabolized to water-soluble compounds that facilitate their subsequent biliary or renal excretion. Hepatic elimination of most toxic substances involves cytochrome P450 enzymes (CYP) [5,6] system and UDP-glucuronosyltransferases [7].

### 2.1. ROS and Antioxidant Defense

ROS are produced by normal cellular metabolism. The main source of endogenous ROS in the liver, as well as in other organs, is oxidative phosphorylation in the mitochondrial electron transfer chain and nicotinamide adenine dinucleotide phosphate NADPH oxidase enzymes (NOX). Mitochondrial ROS generation will depend on the metabolic rate, although the presence of toxic compounds and their transformation by CYP can sometimes be another source of cytosolic ROS, associated with the consumption of NADPH by CYP [8] ROS is a physiological consequence not only of normal cell function but also of the presence of unpaired electrons in free radicals, which gives them high reactivity and can cause damage to other cellular components, such as proteins, lipids, and DNA. An excess of ROS could therefore trigger a state referred to as oxidative stress.

**Figure 1:**
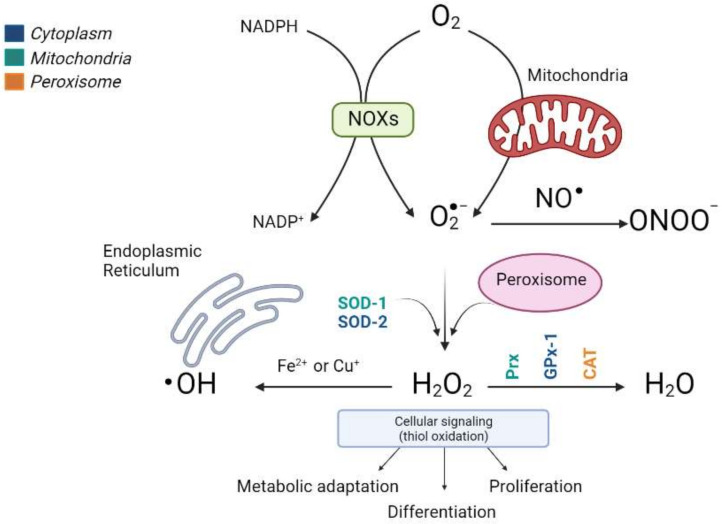
Production scheme of different types of ROS and the antioxidant enzymes involved in their elimination. The main sources of endogenous ROS are oxidative phosphorylation in the mitochondrial electron transfer chain and NOX enzymes. Cytosolic superoxide (O_2_^−^) is quickly converted into hydrogen peroxide (H_2_O_2_) by SOD. H_2_O_2_ oxidizes critical thiols within proteins to regulate vital biological processes, including metabolic adaptation, differentiation, and proliferation, or it can be detoxified in water (H_2_O) by Prx, GPx, and CAT. Moreover, H_2_O_2_ reacts with Fe^2+^ or Cu^2+^ to generate the hydroxyl radical (^•^OH) that causes irreversible oxidative damage to lipids, proteins, and DNA. The different colors indicate the subcellular location of the antioxidant enzymes. (Image created in biorender.com accessed on 19 October 2021).

The most important ROS, which includes radical superoxide (O_2_^−^), non-radical hydrogen peroxide (H_2_O_2_), and hydroxyl radicals (•OH^−^, and the reactive nitrogen species (RNS) that derive from peroxynitrite (ONOO^−^), are the most relevant radical species present in living systems (Figure 1).

Fortunately, and in contrast, liver cells also have potent antioxidant enzymatic and nonenzymatic mechanisms to prevent ROS and repair any damage caused. The antioxidant enzymes include cytosolic and mitochondrial superoxide dismutase (SOD), which eliminates the superoxide ion by converting it into hydrogen peroxide and glutathione peroxidase (GPx), which are involved in detoxifying hydrogen and cellular peroxides for their conversion into oxygen and water, acting in tandem with peroxiredoxins (Prx), thioredoxins (Trx) and glutaredoxins (Grx), and peroxisomal catalase (CAT) (Figure 1). In addition, nonenzymatic molecules such as reduced glutathione (GSH) are present at high concentrations in the liver; vitamin A, vitamin C, vitamin E, bilirubin, ubiquinone, and uric acid remove ROS and restore reduced protein and lipid reserves. Ceruloplasmin and ferritin also help to eliminate the metals that promote oxidative reactions [9,10,11,12].

Alterations in ROS production and/or diminished defense mechanisms can cause serious problems that trigger liver failure [13,14].

When the balance between ROS production and/or antioxidant mechanisms is modified, the onset of oxidative stress leads to cell damage and toxicity and, therefore, multiple pathologies, including hepatic fibrogenesis [15,16,17].

Prolonged fasting produces oxidative stress, increasing hepatic free radical levels and decreasing antioxidant defenses [18,19] Nevertheless, intermittent fasting has also been linked to a reduction in oxidative stress [20,21,22,23,24].

### 2.2. Hepatic Oxidative Stress and Nutritional Status

Oxidative stress may depend on nutritional conditions. Hyperglycemia induces the hyperactivation of NADPH oxidases, increasing oxidative stress [25]. During fasting or calorie restriction, cells are adapted by a metabolic shift in their energy source from glycolysis to oxidative phosphorylation [26,27,28], which requires an increase in mitochondrial oxidative phosphorylation for producing adenosine triphosphate (ATP), and therefore involves elevated ROS production [29].

Many chronic liver diseases are known to be associated with elevated oxidative stress [30]. Thus, the hyperglycemic state that characterizes insulin resistance, diabetes, and obesity [31] could modify cellular redox homeostasis and trigger oxidative stress, mirroring the effect of prolonged fasting. Oxidative stress has been involved in the pathophysiology of several liver diseases. For example, free radicals contribute to the onset and progression of non-alcoholic steatohepatitis (NASH) [32,33], cirrhosis, and liver cancer [34,35]. Mitochondrial ROS promote the presence of other mutations and favor metastatic processes in cancer cells [36].

ROS also operate as signaling molecules in support of normal biological processes and physiological functions. For example, ROS are involved in growth factor signaling, autophagy, hypoxic signaling, immune responses, and stem-cell proliferation and differentiation [10,37,38,39].

## 3. Nutrient Sensors and Oxidative Stress

Nutrient sensors detect changes in nutritional status and suitably adapt an intermediary metabolism to maintain energy and oxidative homeostasis. The following are examples of these sensors: AMPK, mTOR, PASK, and SIRTs.

### 3.1. AMPK and mTOR

AMPK is an energy sensor activated by low energy states or metabolic stress. AMPK activation inhibits anabolic pathways and stimulates catabolic ones to restore the energy balance. AMPK plays a major role in hepatic metabolism [40]. By contrast, mTOR responds to favorable energy states, growth factors, and nutrient-stimulating anabolic processes, as well as cell proliferation and autophagy [41]. In recent years, several studies have also supported its role in the regulation of oxidative stress [42,43].

**Figure 2:**
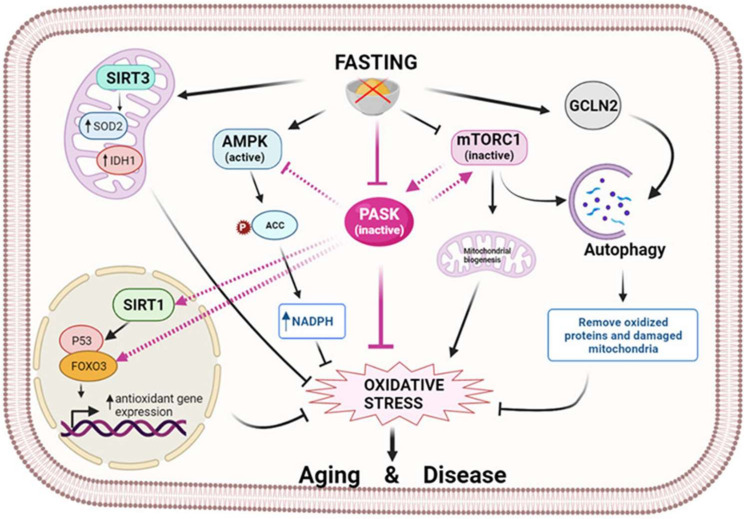
Fasting modulates oxidative stress through nutrient sensors. Fasting initiates a signaling cascade that leads to the activation of antioxidant mechanisms to reduce oxidative stress. Several sirtuins, in particular SIRT1 and SIRT3, are activated by fasting and reduce oxidative stress by controlling antioxidant expression at the transcriptional or post-translational level. In turn, fasting activates AMPK, which prevents oxidative stress by decreasing fatty acid synthesis and increasing the level of NADPH. In parallel, mTOR is inhibited, and GCN2 kinase is activated by fasting, thereby facilitating the autophagy process and the elimination of oxidized proteins and damaged mitochondria. At the center of this scenario is PASK, which fasting keeps inactive, exerting an oxidative stress-reducing effect partly by increasing the antioxidant mechanism. This action could be prompted by the inter-regulation of PASK, AMPK, mTOR, and SIRTs through their activation/deactivation, preventing aging and associated diseases. (Image created in biorender.com accessed on 19 October 2021).

Physiological or pathological conditions, such as hypoxia and glucose deprivation, activate AMPK to promote cellular adaptation for maintaining metabolic and redox homeostasis [44,45]. ROS appear to stimulate AMPK, which promotes mitochondrial biogenesis and the antioxidant defense [46] (Figure 2).

ROS activate AMPK directly or indirectly: activation of both major upstream kinases of AMPK, such as liver kinase B1 (LKB1) and Ca^2+^/calmodulin-dependent protein kinase kinase β (CaMKKβ), and by direct oxidative modification of the AMPKα catalytic subunit [46,47]. ROS induce the S-glutathionylation of AMPKα cysteine residues [48] and could therefore activate AMPK under certain physiological or pathological conditions [49]. This also inhibits the mTORC1 complex through the regulatory-associated protein of TOR (Raptor) phosphorylation, a component of this complex [50], and by phosphorylation of tuberous sclerosis 2 (TSC2), an inhibitor of mTORC1 [51]. This means that the lower activity of the mTOR pathway has also been linked to increased mitochondrial biogenesis and ROS in hematopoietic stem cells [52,53]. The control of protein synthesis depends on the mTOR pathway, stimulated by signals from nutrient glucose and amino acids, while also responding to amino acid starvation, which is detected by general controlled non-repressed (GCN2) kinase. The genetic deletion of GCN2 kinase in intestinal epithelial cells also increases ROS and intestinal inflammation [54] (Figure 2).

### 3.2. PASK

PASK/PASKIN is a serine/threonine kinase containing an N-terminal Per-Arnt-Sim (PAS) domain able to respond to several intracellular parameters, as light, oxygen, and redox state [55,56]. These PAS domains have a well-conserved three-dimensional structure that creates a hydrophobic pocket where small metabolites bind, initiating cellular signaling [57,58,59]. Despite current efforts, the physiological regulators of PASK are still unknown. The PASK activation model proposes that the interaction of a metabolite with the PAS domain terminates its inhibitory function and transient activation occurs, which will subsequently be stabilized by auto or transphosphorylation and may activate/inhibit various substrates [55,58,60,61]. In mammals, PASK responds according to nutritional status by contributing to the regulation of glucose homeostasis, energy metabolism, and oxidative stress [62,63,64,65]. PASK regulates glucagon and insulin secretion [66,67]. Its role in differentiation processes and epigenetic regulation has recently been described [68,69,70].

PASK-deficient mice record an elevated metabolic rate, which has also been confirmed in PASK knockdown myoblast [71] and neuroblastoma cells [72]. PASK is also a critical signaling regulator of AMPK and mTOR pathways in neuroblastoma N2A cells, the hypothalamus, and the liver [72,73]. Meanwhile, PASK deficiency is associated with a reduction in ROS/RNS levels. Nonetheless, the relationship between PASK and ROS production and oxidative stress is still poorly understood. PAS domains are reported to detect intracellular oxygen, redox state, and various metabolites [55]. Moreover, PASK deficiency is associated with the overexpression of hepatic antioxidant enzymes in the basal state and fasting conditions [74] (see Section 4.1) (Figure 2). In addition, PASK deficiency avoids a decrease in the expression of age-related antioxidant enzymes, maintaining ROS/RNS production at a level similar to that of young wild-type (WT) mice. Aged PASK-deficient mice, therefore, record an overall improvement in their antioxidant mechanism and metabolic phenotype (i.e., PASK deficiency blocks the development of glucose intolerance and insulin resistance in aged mice) [75].

### 3.3. Sirtuin Family

The sirtuin family (SIRTs 1–7) consists of nicotinamide adenine dinucleotide (NAD)-dependent histone deacetylases capable of acting on numerous substrates and regulating the activity of chromatin, enzymes, and transcription factors that control antioxidants, ROS, and cellular oxidative stress [76]. The upregulation of SIRT 1 is suggested as an effective therapy against the development of diabetic complications [77].

Studies on calorie restriction report its protective effect, reducing oxidative stress, damage, and extending a lifespan [78,79]. This protective response requires the presence of a member of the sirtuins family. Mitochondrial sirtuin 3 (SIRT3) stimulates SOD2 activity and reduces ROS levels [80]. SIRT3 also induces the mitochondrial glutathione antioxidant system under calorie restriction [81]. SIRT3 is translocated to the mitochondria in response to stress, where it is cleaved and activated [82]. Increased ROS levels also stimulate SIRT3 transcription [78]. SIRT3 modulates the mitochondrial oxidative phosphorylation pathway [83]. Furthermore, SIRT3 regulates the mitochondrial metabolism, and together with other members of the sirtuin family, such as SIRT1, increases the lifespan of experimental animals [84,85]. There is further evidence to suggest that SIRT3 increases longevity in humans [86]. SIRT1 also regulates cellular redox homeostasis through the deacetylation of the main longevity factor forkhead box O-3a (FoxO3a) [87,88], which controls the expression of certain antioxidant genes [89] (Figure 2).

## 4. Potential Role of PASK and Exendin-4/GLP-1 in Therapy

Mutations in the human *PASK* gene have been reported in metabolic diseases such as early-onset diabetes [63]. However, a lower expression of PASK has been reported in pancreatic islets from type 2 diabetic patients [66]. PASK has also been proposed as a possible target in the treatment of diabetes and obesity [71,90].

Exendin-4 (an analog of GLP-1) is used in the clinical management of type 2 diabetes by acting on glucose-stimulated insulin secretion, gastric emptying, and appetite suppression [91]. Besides these effects, exendin-4 is reported to reduce liver lipids, plasma alanine transaminase (ALT), cholesterol, and triglycerides in both humans and mice [92,93,94,95].

### 4.1. PASK Deficiency Reduces Hepatic Oxidative Stress

PASK-deficient mice are protected against obesity and the insulin resistance induced by an HFD [71,96,97]. PASK regulates energy metabolism and glucose homeostasis, especially when adapting to fasting and feeding. Hepatic PASK expression is altered by an HFD [97]. Additionally, PASK deficiency improves the deleterious effects of an HFD, such as the overexpression of hepatic genes that occurs in HFD-fed mice. In addition, PASK deficiency restores glucose tolerance and insulin sensitivity in mice under an HFD, maintaining body weight and serum lipid parameters within the physiological range [97].

High levels of ROS are associated with insulin resistance, type 2 diabetes, and obesity [98]. The role of PASK in hepatic oxidative stress has been investigated under basal and fasting conditions in order to observe the liver’s adaptive response.

The adaptation to energy requirements under prolonged fasting depends on mitochondrial biogenesis. Peroxisome proliferator-activated receptor gamma coactivator 1-alpha (PGC1α) promotes cellular adjustment to conditions requiring energy input, enhancing mitochondrial mass [99,100,101]. PGC1α and SIRT1 are coactivators of several transcription factors and nuclear receptors, such as nuclear respiratory factors (NRFs), peroxisome proliferator-activated receptors (PPARs), and estrogen-related receptors (ERRs).

The expression of coactivator *Ppargc1a* transcription factors such as *Pparg* and *FoxO3a*, and activators such as deacetylase *Sirt1*, are overexpressed under basal conditions in PASK-deficient mice. Furthermore, the SIRT1 subcellular location is mainly nuclear in PASK-deficient mice [74]. Previous data have shown that an increase in nuclear SIRT1 activity, without changes in protein levels, positively correlates with an increased expression of genes regulated by PGC1α [102]. In contrast, the downregulation of PGC1α in obesity has been related to mitochondrial damage and decreased mass [103].

NRF2 (nuclear factor erythroid 2-related factor 2) is considered the major regulator of the cellular redox balance [104,105,106]. NRF2 is usually degraded by the proteasome in the absence of oxidative stress. Nevertheless, NRF2 is translocated into the nucleus when there is an increase in such stress, inducing the expression of several genes coding to glutamate-cysteine ligase (GCLm) and heme oxygenase (HO1) [107,108]. NRF2 activation could be regulated positively by phosphorylation [109,110]. PASK deficiency, therefore, promotes extracellular signal-regulated kinases 1/2 (ERK1/2) overactivation [74], and likewise, the PI3K-AKT pathway is over-activated [97,111]. In turn, PASK deficiency increases the expression of proteins and mRNAs coding to NRF2, GCLm, and HO1 under fasting conditions. These results are consistent with the data reporting that AKT activation decreases glycogen synthase kinase-3 beta GSK3β activity and increases NRF2 nuclear translocation [112], which promotes NRF1 expression and activates mitochondrial biogenesis and antioxidant cellular defenses [113].

Both AMPK activation and elevated SIRT1 under fasting conditions are reported to stimulate FoxO3a nuclear translocation and transcriptional activity [89,114]. Interestingly, PASK deficiency increases the expression of *FoxO3a* under both basal and fasting conditions, as well as the nuclear location of SIRT1 and AMPK activation [74].

PGC1α induces the expression of antioxidant enzymes such as SOD and GPx [115,116,117]. Accordingly, PASK-deficient mice overexpress the hepatic genes coding to antioxidant enzymes GPx and MnSOD in the basal state and also increase the expression in response to fasting of genes coding to MnSOD, Cu/ZnSOD, GPx, GCLm, and HO1 while slightly increasing the *Cat* gene. PASK deficiency is therefore associated with both a reduction in ROS/RNS and slightly higher MnSOD activity under basal conditions [74,75].

Mitophagy has been associated with the FoxO3a transcription factor that controls phosphatase and tensin homolog (PTEN)-induced kinase 1 (PINK1) expression [118]. PASK deficiency also improves the expression of PINK1 involved in cell survival and mitophagy, respectively [74]. In addition, the overactivation of the MAPK pathway seems to maintain a regenerative state. All these effects of PASK deficiency are interesting for states that promote an increase in oxidative stress, such as aging, diabetes, and obesity. Here we have described new evidence in this field, whereby PASK blocking is a powerful promotor of antioxidant mechanisms for preventing oxidative stress in the liver.

### 4.2. GLP-1 Role in Oxidative Stress

GLP-1 derives by post-translational processing from the proglucagon molecule in the intestine and brain [119,120,121,122]. GLP-1 is an incretin released by intestinal L-cells in response to feeding, prompting insulinotropic and glucagonostatic actions from pancreatic beta-cells, potentiating the secretion of insulin, and inhibiting that of glucagon, maintaining glucose homeostasis [123]. Furthermore, GLP-1 records other beneficial actions, such as promoting the proliferation and neogenesis of the pancreatic β-cell [124] and its anorectic properties [125,126,127]. Nevertheless, blood GLP-1 activity is limited by the short half-life due to the action of dipeptidyl-peptidase IV protease [91]. Thus GLP-1 receptor agonists (e.g., exendin-4 and liraglutide) that are more stable and resistant to proteases are used as a therapeutic option in the treatment of type 2 diabetes, based on their glucoregulatory and anorectic actions in mice and humans [91,128,129].

The GLP-1 analog exendin-4 has therefore been used for the clinical treatment of type 2 diabetes [109]. Oral semaglutide (a human analog of GLP-1) will be the first GLP-1 receptor agonist in tablet form, currently in late-stage development, for the treatment of type 2 diabetes. Cardiovascular compatibility has already been confirmed [128].

Exendin-4 has been used since 2005 not only for the treatment of type 2 diabetes but also for hepatic steatosis and non-alcoholic steatohepatitis both in animals and in humans [130].

GLP-1/exendin-4 treatments have been associated with reduced oxidative stress. For example, antioxidant enzymes (SOD, glutathione reductase, CAT, and GPx), as well as glutathione levels, are improved, while other stress markers (lipid peroxidation and nonenzymatic glycosylated proteins) are reduced [95,131].

### 4.3. Evidence for Exendin-4/GLP-1 and PASK Interplay

**Figure 3:**
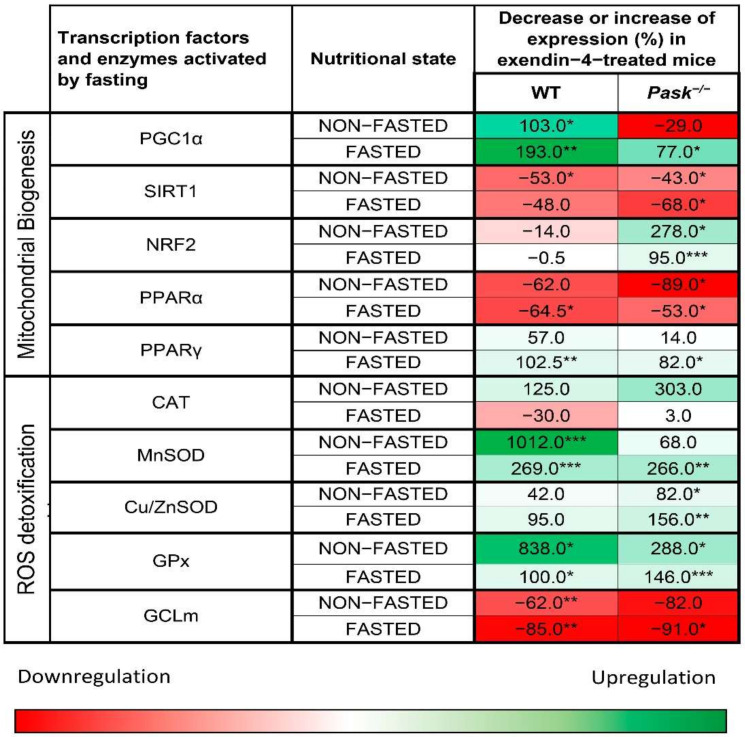
Effect of exendin-4 on the gene expression of hepatic transcription factors involved in oxidative stress and antioxidant enzymes. The animals used were 10- to 16-week-old male mice (25–30 g) C57Bl/6J wild-type (WT) and PASK-defective (*Pask^−^*^/*−*^) back-crossed into C57Bl/6 for at least 13 generations. The animals were fed *ad libitum* with a standard pellet diet (non-fasted) or fasted for 48 h (fasted). Some animals were treated subcutaneously with exendin-4 (250 ng/100 g body weight, Bachem) for three hours. *n =* 4–5 animals per condition. A two-tailed paired Student’s *t*-test was used to analyze the significant differences between exendin-treated mice versus untreated ones. * *p* < 0.05; ** *p* < 0.01 *** *p* < 0.001 untreated vs. exendin-4 treatment. For more details, see Supplementary Materials.

An interesting interplay between PASK and exendin-4/GLP-1 has previously been observed. Thus, PASK deficiency alters certain exendin-4/GLP-1 anorexigenic effects [73]. Likewise, PASK and exendin-4/GLP-1 may control glucose transport and glycogen storage, which are key processes for liver metabolism [132]. Exendin-4 treatment, therefore, blocks hepatic *Pask* expression under both fasting and feeding conditions [132]. The PI3K-AKT pathway is over-activated in PASK-deficient mice [77,91], and exendin-4 treatment decreases AKT activation in a basal state, while no effect has been observed under fasted conditions [132]. This could regulate GSK3 phosphorylation and activity. GSK3 phosphorylates NRF2 creating a recognition motif that promotes the proteasomal degradation of NRF2, independently of the Kelch-like ECH-associated protein 1 (KEAP1) [133]. We have verified the combination of exendin-4 treatment and PASK deficiency in oxidative stress under basal and fasting conditions (unpublished data, see Supplementary Materials). The combination of exendin-4 treatment and the PASK deficiency effect has been studied in relation to the gene expression of certain coactivators, transcription factors, and nuclear receptors involved in mitochondrial biogenesis: *Ppargc1a* encoding PGC1α, *Sirt1, Nrf2*, *Ppara*, and *Pparg*. As well as the expression of the genes coding to ROS detoxification mechanism: CAT, SOD: MnSOD, mainly mitochondrial and Cu/ZnSOD located in cytosol, GPx, and GCLm (Figure 3 and Supplementary Materials).

Exendin-4 treatment regulates oxidative stress both dependently and independently of PASK. For example, the upregulation of *Nrf2* and *Cu/ZnSod* expression by exendin-4 is PASK-dependent, as the inhibition of PASK is needed to increase the expression of these genes by exendin-4 (Figure 3). In turn, exendin-4 increases the gene expression of both *Ppargc1a* in fasting mice and of some antioxidant enzyme genes (i.e., *GPx* and *MnSod*). In these cases, the induction is independent of PASK, as the regulation by exendin-4 occurs in both WT and PASK-deficient mice (Figure 3). These results have been confirmed by the exendin-4 effect on ROS/RNS liver content in vivo. The presence of exendin-4 decreases the percentage (−5.17 ± 0.089) of ROS/RNS content under basal conditions in WT mice, while no effect has been detected in PASK-deficient mice. In contrast, exendin-4 treatment is more effective under fasting conditions when the inactivation of PASK is also included, diminishing the percentage (−10.04 ± 0.38) of ROS/RNS content compared to WT. Exendin-4 treatment has also been reported to increase the *Nrf2* expression associated with a decrease in lipid peroxidation [95,134] and raise GSH levels [135].

These findings suggest that PASK inhibition and exendin-4 treatment might help to promote antioxidant responses to control hepatic oxidative stress and avoid and prevent their harmful effects. According to these results, the use of pharmacologic PASK inhibitors restores many of the hepatic deleterious metabolic consequences associated with NASH [90]. Likewise, exendin-4 is reported to reduce liver fat in obese type 2 diabetic patients [92]. Exendin-4 treatment also reduces hepatic steatosis and an oxidative stress marker in ob/ob mice [136,137].

## 5. Conclusions

The liver is the main coordinator of energy metabolism, performing a wide range of metabolic functions. Its high metabolic activity logically leads to the production of ROS, which in turn is balanced by hepatic antioxidant mechanisms. Nevertheless, both hepatic antioxidant systems and ROS production are disturbed by long fasting, leading to oxidative stress. During prolonged fasting, changes occur not only in the regulation of the hepatic enzymes involved in carbohydrate, lipid, and protein metabolism but also in the genes related to oxidative stress and in antioxidant genes and proteins.

PASK is a metabolic sensor that controls the redox state in the liver and contributes to energy and metabolic homeostasis. In fact, PASK-deficient animals record an altered ATP and ROS production, with an enhanced gene expression of coactivators, transcription factors, and nuclear receptors involved in mitochondrial biogenesis and the expression of antioxidant enzymes. Therefore, PASK blocking promotes the activation of hepatic mechanisms of protection, especially in situations of prolonged fasting, improving cellular redox homeostasis.

In turn, the GLP-1 or its analogs are used as a therapeutic option in the treatment of type 2 diabetes, based on their glucoregulatory and anorectic actions in mice and humans. Furthermore, the GLP-1 analog exendin-4 reduces the hepatic content of ROS, promoting the gene expression of the coactivators, transcription factors, and nuclear receptors involved in mitochondrial biogenesis and the expression of antioxidant enzymes.

There is an interplay between PASK signaling and GLP-1 secretion, as *Pask* expression is blocked by exendin-4 treatment, and reciprocally, PASK deficiency changes the physiological GLP-1 secretory response by intestinal cells after meals.

Our data suggest that it would be interesting to consider PASK inhibition and exendin-4/GLP-1 treatment as a potential therapeutic approach. The use of PASK inhibitors alone or in combination with GLP-1 analogs might help to promote antioxidant responses and avoid and prevent harmful hepatic effects that may be associated with increased oxidative stress.

## Supplementary Materials

The following are available online at https://www.mdpi.com/article/10.3390/antiox10122028/s1, Table S1: Identification of primers used in the Quantitative Real-Time Polymerase Chain Reaction (SYBR GREEN^®^ ASSAY).
