# Supplementary material for: Preventing Oxidative Stress in the Liver: An Opportunity for GLP-1 and/or PASK
Source: Antioxidants (Basel). 2021 Dec 20;10(12):2028. doi: 10.3390/antiox10122028 (PMC8698360; doi:10.3390/antiox10122028)
Supplement: Supplementary file 1 [file antioxidants-10-02028-s001.zip › antioxidants-1447577-supplementary.pdf]

SUPPLEMENTARY MATERIAL

MATERIALS AND METHODS USED IN TABLE S 1

Experimental animals and Exendin-4 treatment

All the procedures involving animals were approved by the appropriate Institutional Review Committee the guidelines for the care of animals specified by the European Community.

The animals used were 10- to 16-week-old males mice (25-30 g) C57Bl/6J wild-type (WT) and PASK-defective (*Pask*$^{-/-}$) back-crossed into C57Bl/6 for at least 13 generations. The animals were housed at a constant temperature (21ºC) on a 12-hour light-dark cycle, with lights on at 8am. Both mice, *Pask*$^{-/-}$ and WT, were kept under standard feeding conditions (*ad libitum*) (non-fasted) or fasted for 48 hours. Some animals (fasted or not; n = 4-5 animals per condition) were treated subcutaneously with exendin-4 (250 ng/100 g body weight, Bachem) for the last three hours. Then, the mice were decapitated and their liver was immediately frozen.

Real-time Polymerase Chain Reaction.

Liver total RNA from WT and *Pask*$^{-/-}$ mice was extracted with TRIzol (Life Technologies, Barcelona, Spain) and cDNA synthesis was developed using the High-capacity cDNA archive kit (Applied Biosystems), using 2 µg of RNA as template, following the manufacturer's instructions. SYBR Green® Assay (Applied Biosystems) was used to quantify the mRNA levels by real-time PCR in a7300HT Fast Real-Time PCR System (Applied Biosystems). The details of the primers and probes are listed in Supplementary Table 1. The PCR conditions were 50 ºC for 2 min, 95 ºC for 10 min, followed by 40 cycles at 95 ºC for 15 s, and 60 ºC for 1 min. *β-actin* housekeeping gene was used for normalization.

Supplementary Table S1. Identification of primers used in the Quantitative Real-Time Polymerase Chain Reaction (SYBR GREEN® ASSAY)

Gene (Protein)	Mouse Forward primer	Mouse Reverse primer
Actb (β-ACTIN)	5'-CTCTCTTCCAGCCTTCCTTC-3'	5'-GGTCTTTACGGATGTCAACG-3'
Cat (CAT)	5'-GAATGGCTATGGTCCACACA-3'	5'-CAAGTTTTTGATGCCCTGGT-3'
Cu/ZnSod (Cu/ZnSOD)	5'-GGTGGTCCACGAGAAACAAG-3'	5'-CAATCACACCACAAGCCAAG-3'
Gclm (GCLm)	5'-TGTGTGATGCCACCAGATTT-3'	5'- GATGATTCCCCTGCTCTTCA-3'
Gpx (GPx)	5'-TGCAATCAGTTCGGACATC-3'	5'-CACCTCGCACTTCTCAAACA-3'
MnSod (MnSOD)	5'-AAGGAGCAAGGTCGCTTACA-3'	5'-ACACATCAATCCCCAGCAGT-3'
Nrf2 (NRF2)	5'-CTACTCCCAGGTTGCCCACA-3'	5'-CGACTCATGGTCATCTACAAATGG-3'
Ppara (PPARa)	5'-TGTTTGTGGCTGCTATAATTT-3'	5'-GCAACTTCTCAATGTAGCCTA-3'
Pparg (PPARg)	5'-GTGCCAGTTTGCATCCGTAGA-3'	5'-GGCCAGCATCGTGTAGATGA-3'
Ppargc1a (PGC1a)	5'-ATGTGTCGCCTTCTTGCTCT-3'	5'-ATCTACTGCCTGGGGACCTT-3'
Sirt1 (SIRT1)	5'-TTGTGAAGCTGTTCGTGGAG-3'	5'-GGCGTGGAGGTTTTTCAGTA-3'

Statistical analyses

Data were presented as percentage of descent or increase in the mRNA levels comparing exendin-4 treated mice respect to non-treated. Differences between groups were first tested with three- way ANOVA, followed by pairwise t-test comparisons. A two-tailed paired Student's t-test was used to analyze the significative differences between exendin-treated mice versus untreated ones. P-values >0.05 were considered statistically significant.
